# Dietary Polysaccharides in Skin Health: Structure–Function Relationships and Implications for Nutritional Dermatology

**DOI:** 10.3390/nu18121838

**Published:** 2026-06-06

**Authors:** Li Zhao, Zhenzhi Chen, Yujie Sun, Ke Jia, Yunjia Liu, Ping Li

**Affiliations:** 1 Graduate School, Beijing University of Chinese Medicine, Beijing 100029, China; 2 Beijing Institute of Traditional Chinese Medicine, Capital Medical University, Beijing 100069, China

**Keywords:** dietary polysaccharides, structure–function relationship, skin health, atopic dermatitis, psoriasis, photoaging, microbiota, immune regulation

## Abstract

Skin health depends on the coordinated maintenance of barrier integrity, immune homeostasis, redox balance, microbial ecology, and systemic metabolic status. Among dietary constituents, polysaccharides have attracted increasing attention because they represent a structurally heterogeneous class of complex carbohydrates whose biological behavior is shaped by molecular weight, monosaccharide composition, glycosidic linkage patterns, branching, higher-order conformation, and physicochemical properties. However, many current skin-related studies remain primarily phenomenon-driven, with insufficient attention to how specific structural features influence biological function and dermatologic relevance. From a structure–function perspective, key structural features of dietary polysaccharides may influence several skin-relevant biological processes, including microbiota-associated signaling, immune regulation, barrier homeostasis, oxidative balance, and extracellular matrix protection. The relevance of these structure-linked functions differs across dermatologic contexts: it appears most direct in photoaging, more conditional in atopic dermatitis, and relatively indirect in psoriasis, whereas wound-repair-related settings are less closely aligned with strict dietary relevance. Current evidence therefore supports structure–function associations more strongly than direct associations between specific structural features and dermatologic outcomes. Dietary polysaccharides are not functionally interchangeable in skin-related contexts, and their skin-related effects depend on structural background, disease setting, and mode of application. Where non-dietary evidence is discussed, it serves primarily as mechanistic or translational contextualization rather than as a basis for nutritional recommendation. Clarifying these relationships may support future mechanistic research and facilitate more rational nutritional applications of dietary polysaccharides in skin health.

## 1. Introduction

Skin health is maintained through the coordinated balance of epidermal barrier integrity, immune homeostasis, redox regulation, microbial ecology, and systemic metabolic status [1]. Accordingly, dermatologic conditions such as atopic dermatitis (AD), psoriasis, photoaging, and impaired wound repair involve disrupted interactions between the skin and broader biological networks, rather than solely localized cutaneous abnormalities. Recent studies in nutritional dermatology increasingly suggest that diet may influence both skin homeostasis and disease susceptibility, supporting the view that nutritional components can actively modulate skin-associated biological pathways [2,3].

Among dietary constituents, polysaccharides have attracted growing attention because they represent a structurally heterogeneous class of complex carbohydrates whose biological behavior is influenced by molecular weight, monosaccharide composition, glycosidic linkages, degree of branching, higher-order conformation, and physicochemical properties such as solubility and viscosity [4]. These structural features are increasingly recognized as important determinants of fermentability, receptor interaction, immune modulation, and antioxidant activity. Food-derived and non-starch polysaccharides have also been associated with skin-relevant biological effects, including the regulation of inflammation, support of barrier function, and protection against oxidative stress [5,6]. For example, certain fermentable polysaccharides may modulate skin inflammation through microbiota-derived short-chain fatty acid signaling [2,3,7], whereas some higher-molecular-weight polysaccharides have been linked to barrier-supportive or immunomodulatory activities [4,8]. However, many current studies in skin-related settings remain primarily phenomenon-driven, with relatively few studies examining how specific structural features shape biological behavior and dermatologic relevance.

Existing reviews have mainly discussed polysaccharides in relation to broad therapeutic potential, immune regulation, or microbiota-associated pathways [9]. In contrast, a synthesis specifically centered on dietary polysaccharides, structural characteristics, and dermatologic relevance remains limited. The present review therefore focuses on dietary polysaccharides within nutritional dermatology, with particular emphasis on orally relevant and food-derived polysaccharides. Key structural features are discussed in relation to skin-relevant biological modules, including microbiota-associated signaling, immune regulation, barrier homeostasis, oxidative balance, and extracellular matrix (ECM) preservation, while comparing how these structure-linked functions differ in relevance across representative dermatologic contexts, particularly AD, psoriasis, and photoaging. Although some studies extend beyond strict nutritional use into topical, formulation-based, or biomaterial-enabled settings, such evidence is discussed only as a limited translational extension.

Overall, this review provides a structure-guided perspective on how dietary polysaccharides may influence skin biology, while highlighting differences in mechanistic relevance across representative dermatologic contexts.

## 2. Dietary Polysaccharides: Definition, Scope, and Structural Basis

Dietary polysaccharides primarily refer to food-derived or edible-source polysaccharides that are naturally consumed as part of the human diet and that possess nutritional or biofunctional relevance to host physiology. This scope includes starch-derived fractions such as resistant starch, non-starch polysaccharides commonly regarded as dietary fibers, and selected polysaccharides derived from edible fungi and edible algae [7,8]. Increasing evidence suggests that these polysaccharides function not only as structural food components, but also as bioactive macromolecules capable of influencing host metabolism, microbial ecology, and immune homeostasis [10,11]. Reviews of fruit polysaccharides and pectic polysaccharides likewise indicate that food source alone does not fully explain biological activity, because polysaccharides derived from different edible matrices may differ substantially in structural organization and functional behavior [12,13]. The discussion therefore emphasizes dietary polysaccharides with clear nutritional relevance, whereas non-dietary studies are included only as limited mechanistic or translational context.

Dietary polysaccharides can be broadly divided into several major classes. One major group includes starch and resistant starch, which are widely distributed in cereals, legumes, and tubers. Unlike digestible starch, resistant starch escapes complete digestion in the upper gastrointestinal tract and behaves more similarly to fermentable dietary fiber in the colon [7,14]. A second major group consists of non-starch polysaccharides, including pectin, inulin-type fructans, β-glucans, arabinoxylans, and galactomannans, which are widely found in fruits, vegetables, grains, and legumes [8,13]. In addition, polysaccharides from edible mushrooms and edible algae also represent important food-derived glycans with growing biofunctional interest [11,15]. These classes differ not only in biological origin, but also in molecular architecture, charge characteristics, and physicochemical behavior, all of which may influence biological activity [4].

Current evidence indicates that the biological activity of dietary polysaccharides depends strongly on structural context. Molecular weight, monosaccharide composition, glycosidic linkage patterns, degree of branching, higher-order conformation, and physicochemical properties such as solubility, viscosity, charge density, and gel-forming capacity have all been associated with differences in fermentability, receptor interaction, immune modulation, and related biological effects [4,16]. Importantly, these variables rarely act independently. Instead, the biological behavior of a dietary polysaccharide usually reflects the combined influence of multiple structural features, together with preparation methods and the biological system under investigation [16,17]. Structural characteristics may therefore influence how polysaccharides behave during gastrointestinal transit, interact with gut microbiota, and modulate host-associated processes such as metabolite production, immune signaling, antioxidant responses, and barrier-related functions.

Taken together, dietary polysaccharides are not functionally interchangeable. Their biological effects are shaped by structural background, disease context, and mode of application. The major structural variables discussed in this section and their associated biological functions are summarized in Table 1.

## 3. Structure–Function Relationships: Biological Modules Relevant to Skin

Structure–function relationships in dietary polysaccharides are context-dependent rather than strictly linear. The biological effects of a given structural feature usually reflect interactions among multiple structural variables, preparation methods, and biological systems rather than isolated parameters alone.

### 3.1. Molecular Weight, Fermentability, and Microbiota-Derived Signaling

Among the structural variables governing polysaccharide function, molecular weight is frequently linked to fermentability in comparative studies, although its biological effects are context-dependent rather than strictly linear [4]. In general, lower-molecular-weight fractions are more soluble and more accessible to microbial glycosidases, which may favor faster degradation and broader substrate utilization. However, recent reviews and comparative studies indicate that the effect of molecular weight depends strongly on the accompanying structural background, including branching, linkage pattern, and conformation. Accordingly, fermentability is shaped by the combined effects of molecular weight, branching, linkage pattern, and conformation.

This principle is supported by representative food-derived polysaccharide studies. In *Momordica charantia* (bitter melon), recent digestion–fermentation analyses have shown that structurally distinct polysaccharide fractions differ in stability during simulated digestion and in subsequent prebiotic behavior during in vitro fermentation, indicating that even within a single edible source, structural variation can substantially alter downstream microbial handling [18]. Similarly, β-1,6-glucan from *Pleurotus eryngii* has been shown to reshape gut microbiota while increasing cecal acetate and butyrate and enhancing mucosal T-cell responses, suggesting that the biological consequence of a dietary polysaccharide partly depends on how its structure is processed within the microbial ecosystem rather than on source identity alone [19].

The underlying mechanisms also depend on how gut microbes process structurally distinct polysaccharides. Members of the genus *Bacteroides* deploy highly specialized polysaccharide utilization loci to sense, bind, and degrade structurally distinct glycans, meaning that different polysaccharide architectures can activate distinct microbial degradation pathways rather than being processed as equivalent substrates [20]. Whereas *Bacteroides* species often initiate the degradation cascade by sensing and cleaving complex glycans, the metabolic fate of the released fragments depends heavily on cross-feeding networks involving specialized fermenters. In this context, dominant butyrate producers such as *Faecalibacterium prausnitzii* are particularly relevant, because their carbohydrate-use capacity helps determine whether glycan degradation is translated into butyrate-rich metabolic output [21].

The downstream consequences are especially important because fermentation-derived short-chain fatty acids are not passive by-products. Butyrate, in particular, has repeatedly been shown to support epithelial barrier integrity and modulate immune polarization, including the suppression of Th17 differentiation and promotion of regulatory T cell-associated responses, partly through histone deacetylase inhibition and related immunometabolic mechanisms [22,23]. These findings link structure-associated fermentability with host inflammatory regulation.

At the same time, fermentation alone does not fully explain the biological effects of dietary polysaccharides. High-molecular-weight, highly viscous β-glucans provide a representative example. Although these polymers may be less readily fermented than smaller fractions, they can still exert important biological effects through their physicochemical behavior in the upper gut. Experimental studies in barley have shown that high-β-glucan intake increases L-cell number and glucagon-like peptide-1 secretion [24].

Microbiota-derived metabolites and viscosity-dependent gut effects may influence the systemic inflammatory background associated with skin disease development. SCFA-mediated expansion of Treg cells and the restraint of Th17-associated inflammation may be more compatible with pathways relevant to Th17-dominant inflammatory conditions such as psoriasis, whereas in AD, where Th2 skewing and intrinsic barrier dysfunction are more prominent, the same route is more likely to provide supportive anti-inflammatory and barrier-stabilizing effects [25,26]. Taken together, these findings suggest that the effects of molecular weight depend largely on how polysaccharides influence microbial fermentation and downstream metabolic signaling rather than on molecular size alone.

### 3.2. Monosaccharide Composition and Selective Biological Interactions

Monosaccharide composition is an important determinant of how dietary polysaccharides interact with gut microbial communities. Current evidence suggests that the biological effects of dietary polysaccharides depend on the combined influence of multiple structural features rather than on a single structural parameter [27]. The relative abundance of glucose, galactose, arabinose, mannose, rhamnose, fucose, or uronic acids contributes to differences in charge behavior, enzymatic accessibility, microbial preference, and host-associated biological activity [4].

At the microbial level, monosaccharide composition helps determine how carbon resources are partitioned across the gut ecosystem. This reflects ecological selectivity rather than simply degradation efficiency. *Bacteroides* spp. and bifidobacteria differ substantially in the glycan-processing systems they deploy, and these differences shape how particular sugar motifs are routed through microbial communities [20,28]. Accordingly, monosaccharide composition contributes to microbial niche partitioning rather than merely to global “fiber utilization.” For example, arabinose-containing structures are frequently associated with bifidobacterial utilization capacity, whereas fucose-containing glycans are consistent with the fucosidase-rich metabolism of *Akkermansia* spp., which are highly specialized for host- and diet-derived fucosylated substrates [29].

Monosaccharide composition may also shape direct host interactions. Mannose-containing motifs, for example, are more likely to intersect with innate immune recognition pathways via receptors such as the mannose receptor (CD206) or through mannose-binding lectin (MBL)-mediated complement activation [30]. Likewise, uronic acid-rich polysaccharides differ from neutral polysaccharides in ionization behavior and intermolecular interactions, which may alter both redox-related properties and host–cell interactions. Accordingly, monosaccharide composition may influence whether dietary polysaccharides interact preferentially with microbial systems, innate immune lectins, or both.

Different monosaccharide compositions may be associated with distinct biological functions relevant to skin health [4,27]. Uronic acid-rich pectic structures may be more commonly associated with barrier-supportive and microbiota-mediated signaling relevant to AD-related biology, whereas mannose-containing glycans may more readily intersect with immune pathways implicated in psoriasis-associated inflammation [4,29,30]. These links remain probabilistic rather than deterministic, but they help explain why “dietary polysaccharide” is not a functionally homogeneous category. Current evidence suggests that monosaccharide composition may influence downstream biological compatibility through effects on microbial utilization and host-associated signaling, rather than serving as a direct predictor of dermatologic outcome [4,27].

### 3.3. Linkage Patterns and Immune Modulation

Glycosidic linkage pattern is a major determinant of how dietary polysaccharides interact with immune recognition systems. The best-established example remains β-glucan, whose β-linkage pattern underlies its recognition by Dectin-1, triggering Syk-dependent signaling and downstream immune signaling pathways involving nuclear factor kappa B (NF-κB) and mitogen-activated protein kinases (MAPK) [31,32]. β-glucan therefore illustrates how linkage patterns can influence immune recognition and signaling.

This linkage-dependent recognition is highly relevant to inflammatory programming because Dectin-1 engagement does not simply trigger a generic immune response; it shapes cytokine output, antigen-presenting cell behavior, and downstream T-cell polarization [33,34]. In psoriasis, this is particularly pertinent because the disease is strongly associated with IL-23/IL-17-dominant circuitry, whereas in AD the inflammatory architecture is canonically more Th2-polarized, albeit with important subtype- and stage-dependent variation [35]. Consequently, certain linkage-defined polysaccharides may engage pathways more closely aligned with psoriasis-like inflammation, whereas in AD their effects may be more consistent with background immunomodulatory activity.

At the same time, β-glucan–Dectin-1 interactions are not the only example of linkage-dependent immune activity. Although this is the best-characterized example, other linkage patterns may also engage distinct pattern-recognition pathways, and the β-glucan model should not be generalized to all dietary polysaccharides with different linkage structures [36,37]. The relevance of linkage pattern to skin health therefore depends on how the induced immune signaling intersects with disease-specific inflammatory pathways, rather than on a direct association between linkage structure and dermatologic outcome.

### 3.4. Branching, Conformation, and Biological Activity

In addition to composition and linkage, degree of branching and higher-order conformation play important roles in shaping the intensity and persistence of biological activity. These variables affect steric accessibility, molecular packing, exposure of active domains, and target recognition, and therefore often help explain why polysaccharides with superficially similar compositions display very different antioxidant, anti-inflammatory, or immunomodulatory behavior [4,16,27]. Reviews of polysaccharide structure–activity relationships indicate that these effects are often non-linear, system-dependent, and not universal [16,27].

A useful example comes from okra pectic polysaccharides, where different degrees of esterification—a parameter that markedly alters chain conformation and intermolecular association—were shown to significantly modify both antioxidant and immunostimulatory activities [38]. These findings suggest that subtle structural rearrangements can alter the biological profile of a polysaccharide even when the overall source remains unchanged, indicating that biological activity depends not only on composition but also on spatial organization [16,38].

Branching and conformation may contribute to differences in antioxidant or anti-inflammatory effects across experimental models. Such variability reflects the fact that structure–activity relationships are often threshold-dependent, variable, or highly sensitive to molecular context [16,27]. For skin health, these structural variables may help explain differences in the intensity and persistence of biological activity across different experimental settings. Accordingly, branching and conformation may influence the strength and persistence of biological responses rather than directly determining dermatologic specificity [16,27].

### 3.5. Physicochemical Properties, Barrier Biology, and Redox Homeostasis

A final major biological module relevant to skin biology concerns physicochemical behavior, especially viscosity, gel formation, water-holding capacity, charge density, and mucosal interaction. In the gut, highly viscous or gel-forming polysaccharides can alter transit, substrate accessibility, mucus interaction, and epithelial exposure, thereby supporting barrier function and reducing systemic inflammatory signals that may reach peripheral tissues [24,39].

Intestinal barrier integrity is tightly linked to systemic inflammatory regulation. SCFAs contribute to this process not only by serving as microbial metabolites, but also by upregulating tight junction proteins such as occludin and claudin-1 and by improving tight-junction assembly, thereby restricting paracellular translocation of luminal endotoxins [40]. Accordingly, barrier-supportive polysaccharides may influence inflammatory skin conditions partly through effects on systemic inflammatory spillover.

A related and overlapping module is redox homeostasis. Structural organization can influence radical-scavenging efficiency and broader antioxidant performance, but again the relationship is not strictly linear. Some low-molecular-weight fractions may exhibit stronger direct antioxidant effects, whereas certain ordered or highly interactive structures may exert broader biological effects through signaling-related mechanisms. This distinction is particularly relevant in skin biology, where oxidative stress contributes not only to inflammation but also to photoaging and ECM degradation.

Experimental work with fermented *Dendrobium officinale* polysaccharides (DOP) is illustrative in this regard: in a UVA-damaged human skin fibroblast model, fermented polysaccharides produced using *Lactobacillus delbrueckii subsp. bulgaricus* were administered at 0.5–2.5 mg/mL during 2 h of UVA exposure and subsequent 24 h incubation, and were associated with enhanced antioxidant capacity and reduced degradation of collagen, elastin, and hyaluronic acid [41]. These findings suggest that structure-sensitive modifications associated with reduced molecular weight and altered structural properties may contribute to skin-related biological effects, while supporting the importance of structure-linked behavior over source identity alone.

Barrier-related and redox-related functions illustrate how physicochemical traits can bridge gastrointestinal events and skin-relevant biology. These properties are especially relevant in conditions where chronic inflammatory background, epithelial stability, and oxidative balance are tightly interconnected. The principal skin-relevant biological modules shaped by structural features of dietary polysaccharides are summarized in Table 2.

### 3.6. Section Synthesis

These modules highlight how different structural features of dietary polysaccharides are associated with distinct aspects of biological activity. Current evidence suggests that the biological effects of dietary polysaccharides arise from the combined influence of multiple structural features rather than from any single descriptor alone [4,15,27]. Molecular weight influences fermentability and metabolic output; monosaccharide composition affects selective microbial and host interactions; linkage patterns contribute to immune recognition; branching and conformation influence the intensity and persistence of biological activity; and physicochemical properties influence barrier-related and redox-related effects [4,15,27,30]. Structurally distinct dietary polysaccharides may therefore exhibit different biological behaviors and dermatologic relevance [4,9,15].

At the same time, the strength of evidence differs substantially across different levels of interpretation. Current evidence more strongly supports structure–function relationships than direct relationships between specific structural signatures and dermatologic outcomes. In particular, controlled studies that systematically vary a single structural parameter—such as molecular weight, branching degree, or linkage pattern—while holding the remaining features relatively constant are still scarce, and human studies incorporating skin-specific outcomes are even more limited. Accordingly, current evidence is more suitable for supporting biologically plausible functional relationships than for direct attribution of dermatologic outcomes to specific structural features.

Current evidence is most consistent in explaining functional diversity and identifying biologically plausible routes to skin relevance. Different dermatologic contexts differ not only in pathophysiology, but also in how directly structure-linked functions relate to disease biology.

## 4. Dermatologic Implications of Structure-Guided Functions

### 4.1. Atopic Dermatitis

In AD, type 2-dominant immune dysregulation, microbial imbalance, and oxidative stress involve several of the functional modules discussed in Section 3 [42,43]. However, the degree to which these effects can be interpreted in structural terms is not uniform across polysaccharide classes. At present, the clearest structure-linked route in AD involves fermentable oligosaccharides and resistant starch, whereas for most complex plant-, fungal-, or marine-derived polysaccharides, the evidence more consistently supports convergent biological effects than defined structural determinants.

#### 4.1.1. Fermentable Oligosaccharides and Resistant Starch: The Most Explicit Structure–Function Route

Among the polysaccharide categories investigated in AD, fermentable oligosaccharides provide the most coherent link between molecular architecture and downstream biological consequence. Fructooligosaccharides such as FOS and kestose are short fructans enriched in β-(2 → 1)-linked fructosyl residues, galactooligosaccharides are short galactosyl oligomers derived from lactose, and xylooligosaccharides (XOS) are based on short β-(1 → 4)-linked xylose chains. Despite chemical differences, these molecules share two functionally important properties: resistance to digestion in the upper gastrointestinal tract and high accessibility to microbial fermentation in the colon [43]. These features facilitate microbiota-dependent signaling, short-chain fatty acid production, and downstream immune modulation.

This logic is consistent with the available AD evidence. Fructo-oligofructose alleviated DNFB-induced AD-like lesions while increasing beneficial genera and fecal SCFAs [44]. XOS was associated with a low-responder phenotype in oxazolone-induced dermatitis together with the enrichment of *Prevotella* [45]. GOS reduced epidermal thickening, inflammatory infiltration, IgE, and cytokine levels while simultaneously reshaping the intestinal microbial ecosystem [46,47]. In infants, prebiotic mixtures containing GOS and FOS were associated with reduced AD incidence or improvement in allergy-related immune markers [48,49]. Collectively, these studies support a role for gut microbial modulation and systemic inflammatory regulation in the effects of fermentable oligosaccharides on AD.

Resistant starch provides an even stronger example of structure–function continuity. Unlike oligosaccharides, resistant starch is defined primarily by digestion-resistant supramolecular organization rather than by a unique monosaccharide composition. Retrograded or otherwise structurally inaccessible starch fractions can escape small-intestinal digestion, reach the colon, and undergo microbial fermentation. In the chickpea-resistant starch study, the material was characterized at the microstructural and crystalline levels, improved AD-like symptoms in a calcipotriol-induced model, lost efficacy after antibiotic treatment, and increased butyrate-associated microbial and metabolic signatures; butyrate then acted through GPR109A and downstream inflammatory signaling [50]. This study links digestion resistance and fermentation compatibility to a defined immunological mechanism involving butyrate, GPR109A, and downstream inflammatory signaling.

Current evidence most strongly supports a link between digestion-resistant structural features and microbiota-dependent biological effects in AD. Even here, however, fine structural variables such as chain length distribution, linkage heterogeneity, or branching pattern are rarely compared systematically within the same disease model. Current evidence therefore supports structure–function relationships more strongly than direct prediction of disease-specific outcomes.

#### 4.1.2. Complex Non-Starch Polysaccharides: Recurrent Functional Effects with Limited Structural Attribution

A second major category comprises structurally more complex non-starch polysaccharides, including DOPs, *Lonicera japonica* polysaccharides, *Houttuynia cordata* polysaccharides, *Ganoderma lucidum* polysaccharides, acidic polysaccharides from red ginseng by-products, and fucoidan [51,52,53,54,55,56,57,58]. Most evidence for these polysaccharides derives from animal and cellular studies with variable treatment durations and concentrations [51,52,53,54,55,56,57,58]. In contrast to fermentable oligosaccharides, these materials commonly differ simultaneously in molecular weight, monosaccharide composition, branching degree, charge density, and conformation [42,55]. Consequently, studies on these polysaccharides have primarily identified recurrent functional outputs rather than resolved structure–function relationships relevant to AD.

Within this group, anti-inflammatory, immune-regulatory, barrier-supportive, and redox-related effects are the most consistent findings. In a DNFB-induced AD mouse model, topical administration of DOP (50–200 mg/kg for 14 days) reduced epidermal thickness, scratching behavior, serum IgE, histamine, TSLP, inflammatory chemokines, and mast cell and CD4-positive cell infiltration, while suppressing MAPK/NF-κB/STAT3 signaling [51]. A later in vivo and in vitro study further showed that orally administered DOP (250–1000 mg/kg during the AD induction period) alleviated oxidative stress and mitochondrial dysfunction, restored mitochondrial dynamics, and inhibited NF-κB activation in AD-associated models [56]. In DNCB-induced AD models, orally administered WLJP-025p from *Lonicera japonica* (30–60 mg/kg during the challenge phase) attenuated AD-like pathology through either p62-dependent Nrf2 activation and NLRP3 degradation or modulation of the Act1/MAPK/NF-κB/AP-1 axis together with barrier recovery [52,59]. Similarly, orally administered Ganoderma-derived GLP-2 (100–200 mg/kg during the experimental intervention period) combined microbiota- and SCFA-related shifts with Th1/Th2 rebalancing, redox improvement, reduced mast cell infiltration, and restoration of barrier-associated readouts in DNCB-treated mice [57]. Collectively, these studies indicate that complex polysaccharides can converge on similar disease-relevant functions, but they do not define which structural variable is causally decisive.

Fucoidan is a sulfated, fucose-rich marine polysaccharide whose biological activity is commonly discussed in relation to sulfation degree, molecular weight, and backbone organization. In a DNCB-induced AD mouse model, orally administered fucoidan from *Cladosiphon okamuranus* (200–800 mg/kg/day for 14 days) reduced epidermal hyperplasia, eosinophil infiltration, serum IgE, and AD-associated cytokine expression, while also improving SCORAD scores [54]. Complementary in vitro experiments further showed that fucoidan (75–600 μg/mL) inhibited mast cell degranulation and reduced IL-4 and histamine release in P815 cells [54]. Together, these findings support fucoidan as a plausible example of a charge-bearing polysaccharide whose structural chemistry may influence immune compatibility in AD, although current studies do not clarify whether sulfation, chain size, or linkage pattern primarily determines biological activity.

β-glucans occupy an intermediate position between structure-informed interpretation and unresolved disease attribution. Yeast and fungal β-glucans are commonly organized around β-(1 → 3) backbones with β-(1 → 6) branching, whereas cereal β-glucans more often display mixed β-(1 → 3)/(1 → 4) linkages. In AD-related models, oral administration of β-1,3/1,6-glucan (0.01 g/kg daily for 7 days) combined with *Lactobacillus plantarum* reduced vasodilation, pruritus, edema, serum histamine, and Th2/Th17-related transcriptional signatures, while enriching butyrate-associated taxa including *Lachnospiraceae*, *Ruminococcaceae*, and *Roseburia* [60]. In a separate HDM-induced Nc/Nga mouse model, oat-derived Synbio-glucan administered as a dietary intervention for 4 weeks and/or topical treatment for 3 weeks improved skin lesion severity and partially restored near-normal skin architecture [61]. These observations suggest that linkage-defined glucan architectures may support immunomodulatory function, although the frequent use of combined or synbiotic preparations limits direct structure-to-disease attribution.

#### 4.1.3. Clinical Signals and Translational Caution

Human evidence remains much more limited than preclinical evidence and provides less structural detail. An uncontrolled pilot study of orally administered *Dendrobium huoshanense* polysaccharide in children with moderate to severe AD reported short-term reductions in SCORAD and several cytokines, but the improvement weakened after discontinuation and the design did not permit mechanistic inference [62]. Randomized infant studies of kestose and GOS/FOS-containing prebiotic mixtures showed lower SCORAD scores or reduced AD incidence, and short-chain galactooligosaccharides (scGOS)/long-chain fructooligosaccharides (lcFOS) supplementation lowered circulating immunoglobulin free light chains in infants at risk for allergy [48,49,63]. In contrast, observational analysis of human milk oligosaccharides did not identify robust associations between individual HMO profiles and AD after correction for multiple testing [64]. Taken together, these data indicate that a translational signal exists, but remains heterogeneous, and the underlying mechanisms are not yet resolved [48,49,62,63,64].

#### 4.1.4. Section Synthesis

Taken together, the AD literature supports graded rather than uniform structure–function relationships. The strongest structure-linked route in this context involves fermentable oligosaccharides and resistant starch, in which defined nondigestibility and microbial accessibility are associated with microbiota-dependent immune modulation and barrier-supportive effects [43,44,45,46,47,48,49,50]. The current translational level remains moderate: dietary relevance is high and early clinical or preventive signals exist, but most evidence is still derived from preclinical or microbiota-centered studies rather than from structure-resolved human investigations. The main unresolved issue is the lack of controlled comparisons of fine structural variants within AD-relevant models, which limits direct attribution from specific structural features to disease-specific outcomes.

### 4.2. Psoriasis

Psoriasis is a chronic immune-mediated inflammatory skin disease characterized by keratinocyte hyperproliferation, aberrant differentiation, and systemic immune activation, in which the IL-23/Th17 axis plays a central pathogenic role [65,66]. Compared with AD, current psoriasis-related evidence provides less direct support for attribution to specific structural features. Instead, broadly defined properties such as nondigestibility and fermentability are more consistently associated with microbiota-mediated regulation, whereas structurally heterogeneous plant- or microbial-derived polysaccharides more often exhibit convergent anti-inflammatory and keratinocyte-regulatory effects [67,68,69,70,71,72,73,74,75,76,77,78,79,80].

#### 4.2.1. Broad Structural Features but Coherent Microbiota-Mediated Functions: Fermentability and Microbiota-Mediated Regulation

A dominant theme in psoriasis-related literature is the close association between gut dysbiosis and disease activity. Reduced microbial diversity, altered microbial composition, impaired barrier function, decreased short-chain fatty acid production, and increased systemic inflammatory signaling have all been implicated in psoriasis pathogenesis [65,66,68,69,71]. These findings provide a mechanistic basis for considering fermentable dietary polysaccharides and prebiotic interventions as relevant modulators of the gut–skin axis.

The most relevant structural features in this context are nondigestibility, microbial accessibility, and fermentation compatibility rather than fine molecular details. These properties enable dietary fibers and prebiotic polysaccharides or oligosaccharides to reshape microbial metabolism and influence immune pathways linked to psoriasis, particularly IL-23/Th17-related inflammation [67,68,70,71]. Clinical and translational studies further suggest that probiotic–prebiotic or synbiotic interventions can improve PASI, quality of life, and inflammatory markers such as LPS, CRP, and IL-1β, although the structural characteristics of the carbohydrate component are often not reported in sufficient detail [81,82].

Thus, in psoriasis, dietary polysaccharides are currently most strongly associated with fermentability-related biological functions rather than with precise structure-specific effects. Current evidence supports a general route linking digestion resistance and microbial utilization to immune modulation, but does not yet allow clear differentiation among different oligosaccharide or polysaccharide structures within the same disease setting.

#### 4.2.2. Structurally Characterized but Biologically Heterogeneous Polysaccharides: Direct Regulation of Keratinocyte and Inflammatory Pathways

A second body of evidence concerns polysaccharides that act more directly on keratinocyte proliferation, inflammatory signaling, and oxidative stress. Unlike microbiota-mediated interventions, these studies often involve cell models or imiquimod-induced psoriasis-like dermatitis models and focus on pathways such as MAPK, NF-κB, PI3K/AKT/mTOR, and oxidative stress-related signaling [72,73,74,75,76,77,78,79,80].

Among them, PSCP from *Saussurea costus* represents one of the relatively better-characterized examples. PSCP was identified as a homogeneous heteropolysaccharide with a molecular weight of approximately 4131 Da and a glucose/fructose-rich repeating structure mainly composed of 1-α-D-Glcp-(-2-β-D-Fruf-1-)23-2-β-D-Fruf [72]. In an IMQ-induced psoriasis-like BALB/c mouse model, intragastric administration of PSCP (200 mg/kg daily for 6 consecutive days) significantly reduced erythema, scaling, epidermal thickening, PASI scores, inflammatory cytokine production, and histopathologic abnormalities, while inhibiting MAPK pathway activation and downstream AP-1 signaling [72]. Because both detailed structural elucidation and anti-psoriatic efficacy were addressed within the same study, PSCP represents one of the relatively few cases in which structural definition and disease-related biological function are directly linked.

β-glucans constitute another structurally meaningful polysaccharide class because their biological activities are closely influenced by glycosidic linkage organization. Yeast- and fungal-derived β-glucans are commonly characterized by β-(1 → 3) backbones with β-(1 → 6) branching, whereas cereal β-glucans more often contain mixed β-(1 → 3)/(1 → 4) linkages. Importantly, structurally distinct β-glucan variants themselves have been comparatively investigated in psoriasis-related disease models. In a mannan-induced psoriasis and psoriatic arthritis-like mouse model, branched 1,3/1,6-β-glucan from *Saccharomyces cerevisiae* (800 μg–5 mg), linear 1,3-β-glucan (curdlan; 3 mg), and linear 1,6-β-glucan (pustulan; 1–3 mg) were each administered intraperitoneally once either 18 days before disease induction, simultaneously with induction, or 1 day after mannan challenge [79]. These structurally distinct β-glucans ameliorated psoriasis- and arthritis-like manifestations through macrophage-dependent immunoregulatory mechanisms involving MMR/CD206-associated pathways and altered resident versus infiltrating macrophage profiles [79]. Because functional differences were examined in relation to defined linkage architectures and branching patterns, β-glucans represent one of the relatively few polysaccharide categories in which structural variants are directly linked to mechanistic differences.

In contrast, many plant-derived polysaccharides are structurally more heterogeneous. In IL-22-, LPS-, and H_2_O_2_-induced psoriasis-like HaCaT keratinocyte models, DOP (typically 0.5 mg/mL for 24 h) inhibited keratinocyte hyperproliferation, inflammatory cytokine expression, and oxidative stress, while suppressing proliferation-associated signaling including p-AKT activation [73]. In an imiquimod-induced psoriasiform dermatitis mouse model, Astragalus polysaccharide (50–200 mg/kg once daily for 6 days) reduced PASI scores, serum TNF-α, IL-1β, and IL-6 levels, as well as macrophage infiltration in skin tissue [80]. Similarly, *Aloe vera* polysaccharide (20–80 μg/mL for 24 h) suppressed TNF-α-induced HaCaT proliferation, inflammatory cytokine production, and NF-κB activation in a dose-dependent manner [75]. Although these findings consistently support anti-proliferative, anti-inflammatory, and anti-oxidative effects, the responsible structural variables remain difficult to isolate because molecular weight, monosaccharide composition, branching, and conformation are not systematically controlled across studies.

Overall, these data support a pattern of functional convergence: structurally diverse polysaccharides can repeatedly suppress keratinocyte-centered inflammation and tissue immune activation in psoriasis models. However, current evidence supports pathway modulation more strongly than structure-specific therapeutic prediction.

#### 4.2.3. Limited Translational Extension Beyond Strict Dietary Relevance

A small number of psoriasis-related studies extend beyond strict dietary use into topical or delivery-oriented systems, including nanofiber membranes, dissolving microneedles, and nanoparticle backbones [74,76,77]. These systems show how polysaccharide structure can support hydration, retention, controlled release, or local tissue modulation. However, these systems are more relevant to formulation engineering than to nutritional action, and are discussed only as limited translational extensions beyond the primary dietary scope.

#### 4.2.4. Section Synthesis

Taken together, structure–function relationships in psoriasis are more indirect and less disease-specific than in AD. The strongest structure-linked association currently involves fermentability-driven, microbiota-mediated regulation, in which nondigestibility and microbial accessibility support modulation of systemic inflammatory tone through the gut–skin axis. The current translational level remains moderate to low: dietary relevance is clear, but much of the available evidence is either system-level and microbiota-mediated or derived from preclinical studies of structurally underresolved polysaccharides. The main unresolved issue is the lack of fine structural comparison within psoriasis-relevant models, which makes disease-specific structural attribution substantially more difficult than in AD.

### 4.3. Photoaging and Skin Health Maintenance

Skin photoaging, primarily induced by chronic ultraviolet (UV) exposure, is characterized by oxidative stress accumulation, DNA damage, ECM degradation, chronic inflammation, and pigmentation disorders. At the molecular level, UV irradiation activates reactive oxygen species (ROS)-mediated signaling cascades, leading to increased matrix metalloproteinases (MMPs), collagen degradation, and dysregulation of pathways such as MAPK, NF-κB, and Nrf2/HO-1 [83].

Compared with inflammatory dermatoses such as AD and psoriasis, photoaging currently provides more direct support for structure–function relationships. This is because a substantial number of studies combine structural characterization—such as molecular weight, monosaccharide composition, and glycosidic linkages—with mechanistic validation, providing more direct evidence for structure–function relationships than is typically available in inflammatory disease settings [84,85,86,87,88].

#### 4.3.1. Structurally Defined Polysaccharides: Linking Molecular Architecture to Anti-Photoaging Activity

A subset of polysaccharides with relatively well-resolved structural features provides important insights into structure-linked photoprotective effects. For instance, a purified polysaccharide (P1) from *Sargassum fusiforme*, composed primarily of mannuronic acid, guluronic acid, and fucose with defined backbone linkages, significantly reduced ROS production, inflammatory cytokines, and MMP expression in UVB-irradiated keratinocytes when administered at 31.25–125 μg/mL for 24 h after UVB irradiation [84]. Similarly, a galactoglucan (PCP-2) from *Pleurotus citrinopileatus*, characterized by (1 → 4)-linked glucopyranosyl and (1 → 4,6)-linked galactopyranosyl residues, tested at 50–200 μg/mL in a UVB-induced zebrafish model for 3 days post-fertilization, attenuated oxidative stress, apoptosis, and cellular senescence via activation of the Nrf2/HO-1 pathway and suppression of MMP-mediated ECM degradation [87].

Plant-derived polysaccharides with detailed structural elucidation also support this relationship. *Pyracantha fortuneana* polysaccharide (31.25–125 μg/mL for 24 h in HaCaT cells; 250–1000 μg/mL for 24 h or 4 days in *C. elegans*) alleviated UVB-induced oxidative stress, apoptosis, MAPK phosphorylation, and tight junction disruption, while also improving lifespan and oxidative stress resistance in nematodes [88]. Likewise, peach gum polysaccharides (PGPs), identified as arabinogalactans with distinct molecular weight fractions, administered at 1.0–1.5 mg/mL for 24 h prior to UVB irradiation in HaCaT cells, inhibited MMP expression and reduced UVB-induced collagen degradation [89].

Taken together, these studies indicate that specific structural features—such as uronic acid content, branching patterns, defined glycosidic linkages, and structural accessibility—can be associated with antioxidant capacity, ECM preservation, and anti-inflammatory activity. However, despite these advances, cross-study comparability remains limited, because structural parameters are often not systematically varied or directly compared within the same experimental framework.

#### 4.3.2. Structurally Heterogeneous Polysaccharides: Functional Convergence on Oxidative Stress and ECM Regulation

In contrast, many polysaccharides investigated for anti-photoaging activity belong to structurally heterogeneous groups, particularly plant- and fungus-derived polysaccharides with variable molecular weight distributions and monosaccharide compositions. Examples include *Lycium barbarum* polysaccharide (LBP; 100 μg/mL for 24 h prior to and during 5-day UVB exposure in HFF-1 cells), which alleviated UVB-induced cellular senescence and oxidative damage via activation of the SIRT3–SOD2 axis [90]; DOP (40–160 μg/mL for 24 h prior to UVB in HaCaT cells), which reduced ROS, apoptosis, and MMP expression while enhancing autophagy and antioxidant defense systems [91]; and lentinan (β-glucan from *Lentinus*; 100–200 μg/mL once daily for 3 days in HDFs), which suppressed NF-κB and MAPK signaling and inhibited senescence-associated pathways (p21/p16 axis) [92].

Additional polysaccharides, such as those derived from *Portulaca oleracea* (low-molecular-weight heteropolysaccharide; 12.5–50 μg/mL for 24 h prior to and after UVB exposure in HaCaT cells [85]), *Alhagi camelorum* (50–200 μg/mL for 24 h pretreatment in HaCaT cells [93]), and *Pholiota nameko* (250 μg/mL for 24 h prior to UVA irradiation in Hs68 cells [94]), similarly demonstrated consistent effects on reducing ROS accumulation, inhibiting apoptosis, and regulating MMP-mediated ECM degradation [85,93,94]. Despite differences in origin and structural complexity, these polysaccharides converge on a limited number of functional pathways, including oxidative stress reduction, MMP suppression, anti-inflammatory signaling, and anti-senescence effects. From a structure-guided perspective, this pattern suggests that while detailed structural determinants remain difficult to isolate, broader physicochemical traits—such as molecular size, solubility, and functional group composition—may still be sufficient to support shared biological effects.

#### 4.3.3. Structure-Modified and Low-Molecular-Weight Polysaccharides: Enhancing Functional Efficiency

An emerging theme in photoaging research is the modification of polysaccharide structure to improve biological activity. For example, degradation of high-molecular-weight polysaccharides into low-molecular-weight fractions significantly enhanced biological activity, as shown for KP-90 (4 kDa) from *Kappaphycus alvarezii*, which at 125–500 μg/mL in vitro (12 h pre-incubation followed by UVB and 24 h culture) and topically at 10–20 mg/mL daily for 7 weeks in vivo reduced MMP expression and improved collagen synthesis [95]. Similarly, fermented DOP (FDOP), characterized by reduced molecular size and improved solubility, administered at 0.5–2.5 mg/mL during UVA exposure and subsequent 24 h incubation, exhibited enhanced activation of the Nrf2/Keap1 pathway and improved protection against UV-induced oxidative damage [41]. Low-molecular-weight fucoidan (LMF) also demonstrated superior anti-photoaging efficacy, applied topically at 0.2–2.0 mg/cm^2^ daily for 15 weeks in UVB-induced mouse models, reducing inflammation, oxidative stress, and wrinkle formation [96]. These findings highlight the importance of molecular weight and structural accessibility as key determinants influencing functional efficiency.

#### 4.3.4. Limited Application-Oriented Extension

Some photoaging studies further extend polysaccharide use into delivery- or formulation-oriented settings, including multilayer emulsions, hydrogels, nanoparticle systems, and host–guest complexes [91,97,98,99,100]. These systems illustrate how polysaccharide macromolecular properties may improve stability, retention, or transdermal delivery. However, these studies are more relevant to delivery engineering than to dietary mechanisms, and are included here only as limited application-oriented extensions.

#### 4.3.5. Section Synthesis

Taken together, structure–function relationships are currently most strongly supported in photoaging. In this context, structurally defined or modified polysaccharides have been associated with antioxidant, anti-inflammatory, anti-senescence, and ECM-protective effects [41,84,87,88,89,95,96]. Most support still comes from cell and animal studies rather than from human dietary intervention studies. A major limitation is the small number of studies directly comparing defined structural variants within the same experimental system, which limits direct assessment of structure–activity relationships.

### 4.4. Wound-Repair-Related Settings as a Limited Translational Extension

Most wound-repair-related studies involving polysaccharides are based on topical hydrogels, dressings, injectable systems, or other biomaterial-enabled applications rather than oral or nutritionally relevant interventions [101,102,103,104,105,106,107,108,109,110,111,112,113,114,115,116]. Current evidence therefore relates primarily to application-oriented biomaterial functions rather than dietary polysaccharide use.

A limited number of studies suggest that structural modification or molecular-weight reduction may alter repair-related bioactivity. For example, octanoyl-modified sulfated galactans from *Gracilaria fisheri* improved fibroblast migration and wound closure more effectively than related fractions [101], while low-molecular-weight polysaccharides from *Enteromorpha prolifera* showed enhanced anti-inflammatory activity and accelerated wound healing [112]. Fucoidan has also been reported to promote angiogenesis and repair through the AKT/Nrf2/HIF-1α pathway [107]. These findings indicate that structural variables such as molecular size, chemical substitution, and charge-related properties may influence wound-relevant functions. However, the dominant evidence in this area remains application-driven, with polysaccharides contributing mainly through hydration, adhesion, gelation, porosity, injectability, and sustained release in repair-oriented systems [102,103,104,105,108,109,113,115].

Accordingly, current evidence in wound-repair-related settings is more informative for application-oriented functionality than for nutritionally relevant dietary action. The relative strength of structure–function relationships across the representative dermatologic settings discussed in this review is summarized in Table 3. Table 4 further organizes representative dietary polysaccharides according to their evidence hierarchy, administration mode, dietary relevance, and translational confidence.

## 5. Cross-Cutting Insights: What a Structure–Function Perspective Can and Cannot Explain

Evidence across AD, psoriasis, photoaging, and wound-repair-related settings indicates functional heterogeneity among dietary polysaccharides. Dermatologic relevance is more consistently associated with specific structural properties and disease context than with botanical, fungal, or algal origin. Current evidence more strongly supports structure–function relationships than direct relationships between structural features and dermatologic outcomes [4,27].

### 5.1. Structure-Linked Functional Compatibility Is Disease-Context Dependent

The strength of structure–function evidence varies across dermatologic contexts. Photoaging currently provides the strongest evidence for structure–function relationships [84,88,92,97]. A relatively large number of studies combine structural characterization with mechanistic validation, making it possible to connect defined molecular features—such as molecular weight, charge, or branching—with antioxidant, anti-inflammatory, anti-senescence, and ECM-protective effects [84,88,92].

In AD, the clearest structure-linked evidence involves fermentable oligosaccharides and resistant starch, where nondigestibility and microbial accessibility are associated with gut microbiota modulation, short-chain fatty acid production, and downstream immune modulation [7,22,23,26,50]. For most complex plant-, fungal-, or marine-derived polysaccharides studied in AD, however, the evidence more consistently supports barrier-supportive, Th2-associated immunomodulatory, and oxidative stress-related effects than specific structure–function relationships [42,51,52,53,54,55,56,57].

In psoriasis, the strongest structure-linked evidence again involves fermentability and microbiota-mediated regulation via the gut–skin axis [65,66,70,71]. Direct anti-psoriatic effects are generally described in terms of pathway inhibition, keratinocyte regulation, or oxidative stress attenuation, while decisive structural determinants remain underresolved [72,73,74,75,76,77,78,79]. Current evidence therefore supports broad structure-related functional associations rather than direct relationships between specific structural features and disease outcomes.

Most wound-repair-related evidence derives from biomaterial-enabled applications rather than nutritionally relevant interventions [102,103,104,105,106,107,108,109,110,111,112,113,114,115]. These studies primarily support material-enabled repair performance rather than nutritionally relevant structure–function relationships.

Different structural features are associated with different dermatologic contexts: fermentability and microbiota accessibility are more strongly associated with AD and psoriasis, whereas structural features linked to ROS attenuation, MMP suppression, and ECM preservation are more commonly associated with photoaging [4,9,27].

### 5.2. What the Framework Can Explain Well

Current evidence supports several recurring structure–function patterns across the literature. Different polysaccharide classes are repeatedly associated with distinct functional effects: fermentable oligosaccharides and resistant starch are most consistently associated with microbiota-mediated signaling and systemic immune modulation [7,22,23,26,50]; sulfated or charged polysaccharides are frequently linked to inflammatory and redox-related pathways [27,102,103,104,105,106,107,108,109,110,111,112,113,114,115]; and β-glucans are more consistently linked to linkage-dependent immunomodulatory effects [72,73,74,75,76,77,78,79].

Current evidence is stronger in photoaging and selected AD-related studies [7,50,84,88,92,97], but remains weaker and more association-based in psoriasis [65,66,70,71,72,73,74,75,76,77,78,79]. These differences indicate that the biological effects of dietary polysaccharides should not be generalized across dermatologic contexts.

Source-based categories alone provide limited structural resolution because they do not distinguish the structural properties associated with biological function [15,27,42,102,103,104,105,106,107,108,109,110,111,112,113,114,115].

### 5.3. What the Framework Still Cannot Explain Sufficiently

Several clear limitations persist. The available literature rarely identifies which specific structural variable drives observed biological effects [12,15]. Molecular weight, monosaccharide composition, branching, glycosidic linkage, sulfation, acetylation, conformation, and physicochemical accessibility often vary simultaneously, complicating attribution of activity to any single feature [12,15]. This limitation is particularly pronounced for complex plant- and fungal-derived polysaccharides [15].

Second, most studies rely on single-preparation and single-model designs [15,27]. Consequently, most studies support descriptive structure–function observations rather than controlled structure–activity relationships [15,27].

Third, dermatologic relevance is frequently inferred from pathway compatibility rather than demonstrated through well-matched disease endpoints [9,26,70]. In inflammatory skin diseases, where barrier dysfunction, immune skewing, microbiota changes, and oxidative stress coexist, it remains difficult to assign disease-level specificity to a single structural feature or functional route [9,26,70].

Together, these observations suggest that current evidence is better suited for probabilistic interpretation than deterministic prediction.

### 5.4. A Graded Rather than Absolute Framework

**Figure 1:**
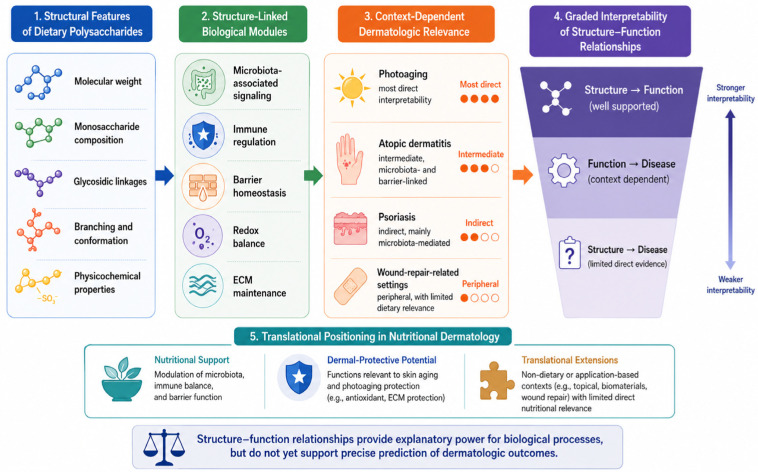
Structure-guided framework of dietary polysaccharides in skin health. Structural features of dietary polysaccharides influence major biological processes relevant to skin health, including microbiota-associated signaling, immune regulation, barrier homeostasis, redox balance, and ECM maintenance. These biological functions show different levels of relevance and interpretability across dermatologic contexts, appearing most direct in photoaging, more conditional in AD, relatively indirect in psoriasis, and more peripheral in wound-repair-related settings. Current evidence supports structure–function relationships more strongly than direct prediction of dermatologic outcomes.

Structure–function relationships in skin-related contexts are graded rather than absolute [9,15]. At the broadest level, structural classes can be associated with dominant functional tendencies [4,15]. At an intermediate level, relatively well-defined polysaccharides permit stronger mechanistic interpretation [50,84,88]. At the most specific level, the literature rarely supports predictive claims that one precisely defined architecture is optimal for a given dermatologic condition [9,15,27]. Dermatologic relevance therefore varies with both structural resolution and disease context [9,15]. This graded framework is summarized in Figure 1.

## 6. Translational Perspectives and Future Directions

Current evidence suggests that dietary polysaccharides are more relevant to supportive dermatologic functions than to stand-alone therapeutic applications. Current evidence more strongly supports context-dependent functional compatibility than precise therapeutic prediction [4,27].

### 6.1. Functional Support, Not Stand-Alone Therapy

Across the conditions discussed here, dietary polysaccharides are most consistently associated with modulation of oxidative stress, microbiota-associated inflammation, barrier dysfunction, ECM degradation, and tissue microenvironment instability. In AD, current evidence most consistently supports microbiota-mediated immune regulation and attenuation of type 2-skewed signaling. In psoriasis, the strongest evidence supports modulation of microbiota-associated systemic inflammation and, for selected polysaccharides, additional effects on keratinocyte-related pathways. In photoaging, polysaccharides may exert more direct dermal-protective effects, particularly when antioxidant and ECM-preserving effects are supported by structural and mechanistic evidence.

However, functional relevance does not necessarily imply therapeutic sufficiency. In chronic inflammatory dermatoses characterized by complex immune dysregulation, tissue remodeling, and genetic susceptibility, dietary polysaccharides are more likely to function as adjunctive or supportive interventions rather than replacements for standard therapies.

### 6.2. Structure-Informed Interpretation of Dietary Polysaccharides

Source-based labels provide limited structural information for dermatologic application. Fermentable oligosaccharides and resistant starch are primarily associated with microbiota-dependent immune modulation through their digestibility profile. Sulfated marine polysaccharides are frequently associated with redox- and inflammation-related functions through their charge-bearing chemistry. β-glucans are not interchangeable by name; linkage architecture and branching pattern matter. Dermatologic relevance is more consistently associated with structure-linked functional properties than with source identity.

### 6.3. Standardization Remains a Major Bottleneck

Lack of standardization remains a major limitation in the current literature. Polysaccharide preparations vary not only by source, but also by extraction method, purification level, molecular-weight distribution, branching pattern, degree of substitution, and residual impurities, often without adequate reporting [9,12,15,17]. This variability, compounded by differences in experimental models and readout panels, makes cross-study comparison unreliable and limits systematic integration of results. More consistent structural characterization and biological testing are necessary for reliable cross-study comparison. Without more rigorous structural documentation and comparable biological testing, current evidence will remain difficult to compare across studies.

### 6.4. Future Research Priorities

Future studies should compare how specific structural features influence functional effects across different disease contexts. This requires comparative designs that systematically vary molecular weight, charge, branching, or fermentability within unified experimental systems. Future studies in AD and psoriasis may benefit from differentiating microbiota-accessible carbohydrate structures and linking them to immune and barrier outcomes. In photoaging, higher-resolution structure–function mapping is already feasible given the field’s relative maturity in integrating structural characterization with functional validation. Future progress depends on generating more structure-resolved and nutritionally relevant evidence across well-defined skin contexts.

## 7. Conclusions

Dietary polysaccharides are not functionally homogeneous in skin-related contexts. Their biological relevance depends primarily on structural features—such as molecular architecture, linkage pattern, branching, and physicochemical behavior—rather than on source category alone, thereby influencing microbiota-mediated signaling, immune regulation, barrier support, redox balance, and ECM preservation. Current evidence most robustly supports structure–function relationships, whereas direct attribution from specific structural features to dermatologic outcomes is limited and context dependent. Structure–function evidence is strongest in photoaging, intermediate in AD, more indirect in psoriasis, and least aligned with strict dietary relevance in wound-repair-related settings. A structure-guided framework provides a means to interpret, compare, and prioritize dietary polysaccharides in nutritional dermatology. Future studies should move beyond phenomenon-driven efficacy studies toward comparative, structure-oriented designs that identify which structural features are compatible with which functional routes in specific skin contexts. Particular priority should be given to orally relevant and nutritionally realistic evidence, while non-dietary studies are primarily informative for mechanistic or translational context rather than direct nutritional recommendation. The next step is to determine which structural attributes confer nutritionally relevant advantages in well-defined skin contexts.

## Figures and Tables

**Table 1:** Key structural variables of dietary polysaccharides and their associated biological functions.

Structural Variable	Representative Examples	Associated Biological Functions	References
Molecular weight	High- and low-molecular-weight β-glucans, pectic polysaccharides, and fruit-derived polysaccharides	Associated with differences in solubility, viscosity, diffusion behavior, fermentability, and target accessibility	[4,16]
Monosaccharide composition	Glucose-, galactose-, arabinose-, mannose-, or uronic acid-rich polysaccharides	Associated with charge-related behavior, microbial utilization preference, and host interaction patterns	[4,12]
Glycosidic linkage pattern	α-linked starches, β-linked glucans, and pectic backbones with distinct linkage architectures	Associated with digestibility, conformational behavior, and, in some cases, receptor recognition	[16,17]
Degree of branching	Linear and branched fruit-, fungal-, or plant-derived polysaccharides	Associated with steric organization, exposure of active domains, and solution behavior	[16,17]
Higher-order conformation	Helical or aggregated conformations in selected polysaccharides	Associated with structural stability, target recognition, and biological responsiveness	[16,17]
Physicochemical properties	Viscous, charged, gel-forming, or water-holding polysaccharides	Associated with gastrointestinal behavior, mucosal interaction, microbial accessibility, and hydration-related performance	[4,8]

**Table 2:** Skin-relevant biological functions associated with structural features of dietary polysaccharides.

Biological Module	Principal Structural Features Implicated	Key Mechanistic Intermediates	Dermatologic Relevance	References
Fermentability and microbiota-derived signaling	Molecular weight; monosaccharide composition; glycosidic linkage; solubility and accessibility	PUL activation; microbial cross-feeding; SCFA production; Treg/Th17 modulation	Inflammatory conditions influenced by gut-derived systemic regulation	[18,19,20,21,22,23,24]
Selective microbial and host interactions	Monosaccharide composition; charge-related features; uronic acid content	Selective microbial utilization; mannose receptor/MBL-related interactions; host–microbe interface effects	Conditions involving microbial dysbiosis or barrier impairment	[20,27,28,29,30]
Immune recognition and inflammatory signaling	Glycosidic linkage pattern; branching; conformation	Dectin-1/Syk; NF-κB; MAPKs; cytokine programming	Immune-mediated inflammatory conditions	[31,32,33,34,35,36,37]
Barrier-associated functions	Viscosity; gel-forming capacity; water-holding ability; charge density	Mucosal interaction; endotoxin restriction; tight-junction support	Barrier-impaired and inflammatory skin conditions	[24,39,40]
Antioxidant and redox-related effects	Branching degree; higher-order conformation; monosaccharide composition; molecular weight	Radical scavenging; Nrf2-related signaling; oxidative stress attenuation	Conditions associated with oxidative stress and ECM degradation	[38,41]

**Table 3:** Context-dependent structure–function relationships across representative dermatologic settings.

Dermatologic Context	Most Relevant Structure-Linked Features	Dominant Functional Route	Most Strongly Supported Structure–Function Relationship	Current Translational Level	Main Unresolved Issue	Dietary Relevance in the Present Review	References
AD	Nondigestibility, fermentability, microbial accessibility; selected linkage-defined classes	Microbiota-mediated immune modulation; barrier-supportive and redox-related effects	Fermentable oligosaccharides and resistant starch, for which defined nondigestibility and microbial accessibility are associated with microbiota-dependent immune modulation and barrier-supportive effects	Moderate; dietary relevance is high and early clinical or preventive signals exist, but most evidence remains preclinical or microbiota-centered	Lack of controlled comparisons of fine structural variants within AD-relevant models	High	[42,43,44,45,46,47,48,49,50,51,52,53,54,55,56,57,58,59,60,61,62,63,64]
Psoriasis	Broad fermentability-related properties; selected linkage-defined or better-characterized polysaccharides	Gut–skin axis modulation; keratinocyte-centered anti-inflammatory and antioxidant effects	Fermentability-driven microbiota-mediated regulation linked to gut–skin axis modulation	Moderate to low; relevant from a dietary perspective, but much evidence remains system-level, microbiota-mediated, or preclinical	Lack of fine structural comparison within psoriasis-relevant models, limiting disease-specific structural attribution	Moderate	[65,66,67,68,69,70,71,72,73,74,75,76,77,78,79,80,81,82]
Photoaging	Molecular weight, linkage patterns, branching, uronic acid content, structural accessibility	Antioxidant, anti-inflammatory, anti-senescence, and ECM-protective effects	Structurally defined or structurally modified polysaccharides linked to antioxidant, anti-inflammatory, anti-senescence, and ECM-protective functions	Moderate; dietary and dermal-protective relevance is stronger than in wound-repair-related settings, but most evidence still comes from cell and animal studies	Limited direct comparison of defined structural variants within unified experimental systems, restricting direct structure–activity relationships	Moderate to high	[41,83,84,85,86,87,88,89,90,91,92,93,94,95,96,97,98,99,100]
Wound-repair-related settings	Molecular-weight reduction, chemical substitution, charge-related properties; physicochemical architecture	Inflammation control, macrophage reprogramming, angiogenesis, and materials-enabled microenvironmental support	Functionally relevant structural modification or physicochemical architecture in repair-oriented systems	Low; most evidence is application-driven rather than nutritionally relevant	Lack of structure–function studies in nutritionally relevant contexts	Low	[101,102,103,104,105,106,107,108,109,110,111,112,113,114,115,116]

**Table 4:** Representative dietary polysaccharides in skin health: structural characteristics, administration mode, dietary relevance, and current evidence.

Polysaccharide/Class	Structural Feature(s) Relevant Here	Representative Treatment Condition	Administration Mode	Skin Context	Strongest Supported Function	Evidence Level	Dietary Relevance	Main Limitation	References
Fructooligosaccharides (FOS)/oligofructose	Short-chain fructans; β-(2 → 1)-linked fructosyl residues; nondigestibility; high fermentability	1.0 g/kg oral administration for 12 days in DNFB-induced AD mice	Oral/dietary	AD	Microbiota modulation; SCFA production; systemic immune modulation	Animal evidence; limited supportive human/preventive data	High	Fine structural comparison within AD models remains limited	[44]
Kestose/GOS-FOS mixtures/scGOS-lcFOS	Short-chain prebiotic oligosaccharides; high microbial accessibility	100–200 mg/kg dietary supplementation for 8 weeks in AD models; 0.8 g/100 mL supplementation in allergy-risk infants	Oral/dietary	AD	Reduced AD incidence/risk markers; microbiota-linked immune support	Clinical/preventive human evidence; limited structure-specific attribution	High	Mixed-prebiotic evidence limits component-specific structural attribution	[46,47,48,49]
Resistant starch (e.g., chickpea resistant starch)	Digestion-resistant supramolecular organization; colonic fermentability	15% resistant starch diet for 14 days in MC903-induced AD mice	Oral/dietary	AD	Butyrate generation; GPR109A-related immune modulation	Animal with mechanistic support	High	Human translational evidence remains limited	[50]
β-glucans (oral dietary preparations)	Linkage-defined glucan architecture; source-dependent β-linkages	0.01 g/kg/day oral β-glucan for 7 days in AD-related models; 2% β-glucan diet for 4 weeks in NC/Nga mice; 0.8 μg–5 mg structurally distinct β-glucans in psoriasis-related models	Oral/dietary; some synbiotic formulations	AD; psoriasis	Immune modulation; microbiota-related support; inflammatory tone reduction	Animal evidence with mechanistic support	Moderate to high	Mixed or synbiotic preparations limit direct attribution	[60,61,79]
Fucoidan (dietary/bioactive edible-source context)	Sulfated, fucose-rich polysaccharide; charge-bearing properties	Typically evaluated at oral or topical doses in preclinical AD and UV-induced photoaging models	Mainly preclinical experimental contexts	AD; photoaging	Anti-inflammatory, antioxidant, ECM-protective, or immune-modulatory effects	Cell and animal evidence	Moderate	Relative contribution of sulfation, chain size, and backbone structure remains unclear	[54,96]
DOP	Structurally heterogeneous plant polysaccharides; variable molecular weight and composition	50–200 mg/kg topical or 250–1000 mg/kg oral in AD mice; 0.125–32 mg/mL for 24 h in HaCaT cells; 2–4 g/day oral for 4 weeks in children with AD	Oral and topical experimental contexts	AD; psoriasis	Anti-inflammatory, antioxidant, barrier- or ECM-supportive effects	Cell and animal evidence; limited pilot human data	Moderate	High structural heterogeneity and unresolved disease-relevant causal features	[51,56,62,73]
PSCP from *Saussurea costus*	Relatively well-characterized heteropolysaccharide; defined molecular weight and repeating features	200 mg/kg/day oral for 6 days in IMQ-induced psoriasis-like mice	Experimental oral administration	Psoriasis	MAPK/AP-1 pathway inhibition; anti-inflammatory and anti-proliferative effects	Animal evidence with mechanistic support	Low to moderate	Stronger as proof-of-concept evidence than as nutritionally realistic evidence	[72]
LBP	Complex plant polysaccharide; heterogeneous composition	~100 μg/mL for 24 h in cell-based photoaging models	Cell-based experimental context	Photoaging	Antioxidant and anti-senescence effects	Cell and animal evidence	Moderate to high	Structure–activity mapping remains incomplete	[90]
Structurally defined photoaging-related polysaccharides (e.g., P1, PCP-2, PPFP, PGPs)	Defined linkage patterns, branching, uronic acid content, and molecular-weight-related features	Typically evaluated at ~30–200 μg/mL for 24 h to 3 days in UV-induced photoaging models	Experimental cell and animal contexts	Photoaging	Antioxidant, anti-inflammatory, and ECM-preserving effects	Cell and animal evidence	Moderate	Usually evaluated as single preparations without cross-variant comparison	[84,87,88,89]
Low-molecular-weight or modified photoaging-related polysaccharides (e.g., KP-90, FDOP, LMF)	Molecular-weight reduction; enhanced accessibility/solubility; structurally modified efficiency	KP-90: 125–500 μg/mL in vitro; topical 10–20 mg/mL daily for 7 weeks in UV-induced photoaging mice	Experimental cell and animal contexts	Photoaging	Enhanced antioxidant efficiency; reduced MMPs; skin-protective effects	Cell and animal evidence	Moderate	Functional improvement is shown, but predictive structure–activity rules remain lacking	[41,95,96]
Wound-repair-related polysaccharide systems	Molecular-weight reduction; substitution; charge-related properties; physicochemical architecture	Typical μg/mL-level scratch assays and topical hydrogel-based wound models	Mostly topical, dressing, hydrogel, or injectable systems	Wound-repair-related settings	Repair-oriented materials performance; inflammation control; angiogenesis support	Biomaterial/animal/mechanistic	Low	Dominated by non-dietary, application-driven evidence	[101,102,103,104,105,106,107,108,109,110,111,112,113,114,115,116]

## Data Availability

No new data were created or analyzed in this study. Data sharing is not applicable to this article.

## Abbreviations

The following abbreviations are used in this manuscript:

AD	Atopic Dermatitis
ECM	Extracellular Matrix
MBL	Mannose-binding lectin
NF-κB	Nuclear factor kappa B
MAPK	Mitogen-activated protein kinase
UV	Ultraviolet
ROS	Reactive Oxygen Species
MMPs	Matrix Metallo Proteinases
PGPs	Peach Gum Polysaccharides
LBP	*Lycium barbarum* polysaccharide
DOP	*Dendrobium officinale* polysaccharide
FDOP	Fermented DOP
LMF	Low-molecular-weight fucoidan
XOS	Xylooligosaccharides

## References

[1] Assaf S., Kelly O. (2024). Nutritional dermatology: Optimizing dietary choices for skin health. Nutrients.

[2] Ryczaj K., Beken B., Akdis C. (2025). Feeding the skin barrier: The impact of macro- and micronutrients on skin barrier function. Clin. Transl. Allergy.

[3] Sharma N., Chaudhary S.M., Khungar N., Aulakh S.K., Idris H., Singh A., Sharma K. (2024). Dietary influences on skin health in common dermatological disorders. Cureus.

[4] Xue H., Tang Y., Zha M., Xie K., Tan J. (2025). The structure–function relationships and interaction between polysaccharides and intestinal microbiota: A review. Int. J. Biol. Macromol..

[5] Zhang Z., Koris A., Csighy A., Yao X., Gu K., Zhang P., Xue B., Ren F., Liu H. (2025). Extraction, structural characteristics, and health benefits of starch, arabinoxylan and β-glucan from Triticeae cereals: A critical review. Int. J. Biol. Macromol..

[6] Strouphauer E., Parke M., Perez-Sanchez A., Tantry E., Katta R. (2023). Functional foods in dermatology. Dermatol. Pract. Concept..

[7] Baptista N.T., Dessalles R., Illner A.K., Ville P., Ribet L., Anton P.M., Durand-Dubief M. (2024). Harnessing the power of resistant starch: A narrative review of its health impact and processing challenges. Front. Nutr..

[8] Alahmari L.A. (2024). Dietary fiber influence on overall health, with an emphasis on CVD, diabetes, obesity, colon cancer, and inflammation. Front. Nutr..

[9] Sun M., Zhang Y., Zhou M., Sui Z. (2025). From nutrition to smart therapy: Food-derived polysaccharides for dermatological applications: A review. Int. J. Biol. Macromol..

[10] Zhang W., Zhang Y., Zhao Y., Li L., Zhang Z., Hettinga K., Yang H., Deng J. (2024). A comprehensive review on dietary polysaccharides as prebiotics, synbiotics, and postbiotics in infant formula and their influences on gut microbiota. Nutrients.

[11] Zhao Q., Jiang Y., Zhao Q., Manzi H.P., Su L., Liu D., Huang X., Long D., Tang Z., Zhang Y. (2023). The benefits of edible mushroom polysaccharides for health and their influence on gut microbiota: A review. Front. Nutr..

[12] Shi L., He Q., Li J., Liu Y., Cao Y., Liu Y., Sun C., Pan Y., Li X., Zhao X. (2024). Polysaccharides in fruits: Biological activities, structures, and structure–activity relationships and influencing factors—A review. Food Chem..

[13] Barrera-Chamorro L., Fernandez-Prior Á., Rivero-Pino F., Montserrat-de la Paz S. (2025). A comprehensive review on the functionality and biological relevance of pectin and its use in the food industry. Carbohydr. Polym..

[14] Wang Z., Wang S., Xu Q., Kong Q., Li F., Lu L., Xu Y., Wei Y. (2023). Synthesis and functions of resistant starch. Adv. Nutr..

[15] Zhang X., Duan Y., Xue J., Chen S., Wang H. (2025). Edible mushroom polysaccharides: Structural characteristics, chemical modification strategies, and structure–activity relationship: A review. Int. J. Biol. Macromol..

[16] Boukid F., Méndez-Albiñana P., Sánchez-Baca A., Villamiel M. (2025). Impact of fiber molecular structure on resistance to digestion using the INFOGEST and rat small intestine extract protocols. Eur. J. Nutr..

[17] Hu Y., Zhang Y., Cui X., Wang D., Hu Y., Wang C. (2024). Structure–function relationship and biological activity of polysaccharides from mulberry leaves: A review. Int. J. Biol. Macromol..

[18] Zhang Y., Chen F., Feng J., Wang F., Xiong L., Wang L., Shen X., Song H. (2025). Simulated digestion and fermentation characteristics of a polysaccharide from *Momordica charantia* L. and the anti-inflammatory activity of its fermentation products. Int. J. Biol. Macromol..

[19] Wang X., Qu Y., Wang Y., Wang X., Xu J., Zhao H., Zheng D., Sun L., Tai G., Zhou Y. (2022). β-1,6-Glucan from *Pleurotus eryngii* modulates immunity and gut microbiota. Front. Immunol..

[20] Feng J., Qian Y., Zhou Z., Ertmer S., Vivas E.I., Lan F., Hamilton J.J., Rey F.E., Anantharaman K., Venturelli O.S. (2022). Polysaccharide utilization loci in *Bacteroides* determine population fitness and community-level interactions. Cell Host Microbe.

[21] Lindstad L.J., Lo G., Leivers S., Lu Z., Michalak L., Pereira G.V., Røhr Å.K., Martens E.C., McKee L.S., Louis P. (2021). Human gut *Faecalibacterium prausnitzii* deploys a highly efficient conserved system to cross-feed on β-mannan-derived oligosaccharides. mBio.

[22] Siddiqui M.T., Cresci G.A.M. (2021). The immunomodulatory functions of butyrate. J. Inflamm. Res..

[23] Xiao X., Hu X., Yao J., Cao W., Zou Z., Wang L., Qin H., Zhong D., Li Y., Xue P. (2023). The role of short-chain fatty acids in inflammatory skin diseases. Front. Microbiol..

[24] Paudel D., Dhungana B., Caffe M., Krishnan P. (2021). A review of health-beneficial properties of oats. Foods.

[25] Yoshida M., Funasaka Y., Saeki H., Yamamoto M., Kanda N. (2023). Dietary fiber inulin improves murine imiquimod-induced psoriasis-like dermatitis. Int. J. Mol. Sci..

[26] Sadowsky R.L., Sulejmani P., Lio P.A. (2023). Atopic dermatitis: Beyond the skin and into the gut. J. Clin. Med..

[27] Chen N., Jiang T., Xu J., Xi W., Shang E., Xiao P., Duan J.A. (2024). The relationship between polysaccharide structure and its antioxidant activity needs to be systematically elucidated. Int. J. Biol. Macromol..

[28] Rakoff-Nahoum S., Coyne M.J., Comstock L.E. (2014). An ecological network of polysaccharide utilization among human intestinal symbionts. Curr. Biol..

[29] Luis A.S., Hansson G.C. (2023). Intestinal mucus and their glycans: A habitat for thriving microbiota. Cell Host Microbe.

[30] Mata-Martínez P., Bergón-Gutiérrez M., Del Fresno C. (2022). Dectin-1 signaling update: New perspectives for trained immunity. Front. Immunol..

[31] de Koning H.D., Rodijk-Olthuis D., van Vlijmen-Willems I.M., Joosten L.A., Netea M.G., Schalkwijk J., Zeeuwen P.L. (2010). A comprehensive analysis of pattern recognition receptors in normal and inflamed human epidermis: Upregulation of Dectin-1 in psoriasis. J. Investig. Dermatol..

[32] Agrawal S., Gupta S., Agrawal A. (2010). Human dendritic cells activated via Dectin-1 are efficient at priming Th17, cytotoxic CD8 T and B cell responses. PLoS ONE.

[33] Tsoni S.V., Brown G.D. (2008). Beta-glucans and Dectin-1. Ann. N. Y. Acad. Sci..

[34] Elder M.J., Webster S.J., Fitzmaurice T.J., Shaunak A.S.D., Steinmetz M., Chee R., Mallat Z., Cohen E.S., Williams D.L., Gaston J.S.H. (2019). Dendritic cell-derived TSLP negatively regulates HIF-1α and IL-1β during Dectin-1 signaling. Front. Immunol..

[35] Sieminska I., Pieniawska M., Grzywa T.M. (2024). The immunology of psoriasis—Current concepts in pathogenesis. Clin. Rev. Allergy Immunol..

[36] Figueiredo R.T., Bittencourt V.C., Lopes L.C., Sassaki G., Barreto-Bergter E. (2012). Toll-like receptors (TLR2 and TLR4) recognize polysaccharides of *Pseudallescheria boydii* cell wall. Carbohydr. Res..

[37] Lu H., Yang Y., Gad E., Wenner C.A., Chang A., Larson E.R., Dang Y., Martzen M., Standish L.J., Disis M.L. (2011). Polysaccharide krestin is a novel TLR2 agonist that mediates inhibition of tumor growth via stimulation of CD8 T cells and NK cells. Clin. Cancer Res..

[38] Li W., Li J., Wang J., He Y., Hu Y.C., Wu D.T., Zou L. (2022). Effects of various degrees of esterification on antioxidant and immunostimulatory activities of okra pectic polysaccharides. Front. Nutr..

[39] Pérez-Reytor D., Puebla C., Karahanian E., García K. (2021). Use of short-chain fatty acids for the recovery of the intestinal epithelial barrier affected by bacterial toxins. Front. Physiol..

[40] Münte E., Hartmann P. (2025). The role of short-chain fatty acids in metabolic dysfunction-associated steatotic liver disease and other metabolic diseases. Biomolecules.

[41] Zhang Y., You S., Wang D., Zhao D., Zhang J., An Q., Li M., Wang C. (2022). Fermented *Dendrobium officinale* polysaccharides protect UVA-induced photoaging of human skin fibroblasts. Food Sci. Nutr..

[42] Pareek A., Behera M., Sahu A., Malani P., Chuturgoon A., Pareek A. (2026). Konjac glucomannan, macrophage polarisation, and atopic dermatitis: Preclinical evidence and translational perspectives—A review. Int. J. Biol. Macromol..

[43] Jeurink P.V., van Esch B.C., Rijnierse A., Garssen J., Knippels L.M. (2013). Mechanisms underlying immune effects of dietary oligosaccharides. Am. J. Clin. Nutr..

[44] Chen S., Tang L., Nie T., Fang M., Cao X. (2023). Fructo-oligofructose ameliorates 2,4-dinitrofluorobenzene-induced atopic dermatitis-like skin lesions and psychiatric comorbidities in mice. J. Sci. Food Agric..

[45] Laigaard A., Krych L., Zachariassen L.F., Ellegaard-Jensen L., Nielsen D.S., Hansen A.K., Hansen C.H.F. (2020). Dietary prebiotics promote intestinal *Prevotella* in association with a low-responding phenotype in a murine oxazolone-induced model of atopic dermatitis. Sci. Rep..

[46] Han K., Ahn Y., Hong K.B., Suh H.J., Yu K.W., Kim H. (2022). Ameliorating the efficacy of galacto-oligosaccharides on ovalbumin-induced allergic dermatitis symptoms in Balb/c mice by regulating Th2 immune response and the ecosystem of gut microbiota. Food Funct..

[47] Tanabe S., Hochi S. (2010). Oral administration of a galactooligosaccharide preparation inhibits development of atopic dermatitis-like skin lesions in NC/Nga mice. Int. J. Mol. Med..

[48] Moro G., Arslanoglu S., Stahl B., Jelinek J., Wahn U., Boehm G. (2006). A mixture of prebiotic oligosaccharides reduces the incidence of atopic dermatitis during the first six months of age. Arch. Dis. Child..

[49] Schouten B., Van Esch B.C.A.M., Kormelink T.G., Moro G.E., Arslanoglu S., Boehm G., Knippels L.M.J., Redegeld F.A., Willemsen L.E.M., Garssen J. (2011). Non-digestible oligosaccharides reduce immunoglobulin free light-chain concentrations in infants at risk for allergy. Pediatr. Allergy Immunol..

[50] Yan Q., Wang W., Fan Z., Wei Y., Yu R., Pan T., Wang N., Lu W., Li B., Fang Z. (2025). Chickpea-resistant starch exhibits bioactive function for alleviating atopic dermatitis via regulating butyrate production. Int. J. Biol. Macromol..

[51] Liao J., Zhao W., Zhang Y., Zou Z., Zhang Q., Chen D., Du B., Li P. (2024). *Dendrobium officinale* Kimura et Migo polysaccharide ameliorates DNFB-induced atopic dermatitis in mice by suppressing MAPK/NF-κB/STAT3 signaling pathways. J. Ethnopharmacol..

[52] Bai X., Rao X., Wang Y., Shen H., Jin X. (2023). A homogeneous *Lonicera japonica* polysaccharide alleviates atopic dermatitis by promoting Nrf2 activation and NLRP3 inflammasome degradation via p62. J. Ethnopharmacol..

[53] Huang R., Zhang W., Hu Y., Xu J., Dong Z., Liu J., Zhou L. (2025). *Houttuynia cordata* polysaccharides ameliorate atopic dermatitis in mice through modulation of skin immune barrier and lipid metabolism. Int. J. Biol. Macromol..

[54] Chen B.-R., Hsu K.-T., Hsu W.-H., Lee B.-H., Li T.-L., Chan Y.-L., Wu C.-J. (2021). Immunomodulation and mechanisms of fucoidan from *Cladosiphon okamuranus* ameliorate atopic dermatitis symptoms. Int. J. Biol. Macromol..

[55] Wang W., Yue W., Shao L., Liu T., Xiao J., Chu Q., Wu S. (2025). Structure characterization and bioactivities of Ampelopsis grossedentata polysaccharides and their anti-inflammatory effect on TNF-α/IFN-γ-induced atopic dermatitis-like HaCaT keratinocytes. Int. J. Biol. Macromol..

[56] Zeng B., Jiang G., Wang C., Zhou H., Zhang Y., Yan Y.-N., Chen Z., Zhang L., Li X., Xie M. (2026). *Dendrobium officinale* polysaccharides alleviate atopic dermatitis in vivo and in vitro through inhibition of inflammation and mitochondrial dysfunction. J. Mol. Histol..

[57] Zhang D., Wei Y., Zhu X., Zong L., Cui M., Li D., Zhang C. (2025). Study on the intervention mechanism of *Ganoderma lucidum* polysaccharides in mice with atopic dermatitis. Food Res. Int..

[58] Kim J., Jang S., Lee C.H., Lee J.Y., Park H., Kim J.H., Lee S., Kim S.H., Park E., Lee K.W. (2019). Beneficial effects on skin health using polysaccharides from red ginseng by-product. J. Food Biochem..

[59] Zhang T., Rao X., Song S., Tian K., Wang Y., Wang C., Bai X., Liu P. (2024). WLJP-025p, a homogeneous *Lonicera japonica* polysaccharide, attenuates atopic dermatitis by regulating the MAPK/NF-κB/AP-1 axis via Act1. Int. J. Biol. Macromol..

[60] Kim I.S., Lee S.H., Kwon Y.M., Adhikari B., Kim J.A., Yu D.Y., Kim G.I., Lim J.M., Kim S.H., Lee S.S. (2019). Oral administration of β-glucan and *Lactobacillus plantarum* alleviates atopic dermatitis-like symptoms. J. Microbiol. Biotechnol..

[61] Kim Y.-H., Kang M.S., Kim T.H., Jeong Y., Ahn J.-O., Choi J.H., Chung J.-Y. (2021). Anti-inflammatory and immune modulatory effects of synbio-glucan in an atopic dermatitis mouse model. Nutrients.

[62] Wu K.G., Li T.H., Chen C.J., Cheng H.I., Wang T.Y. (2011). A pilot study evaluating the clinical and immunomodulatory effects of an orally administered extract of *Dendrobium huoshanense* in children with moderate to severe recalcitrant atopic dermatitis. Int. J. Immunopathol. Pharmacol..

[63] Shibata R., Kimura M., Takahashi H., Mikami K., Aiba Y., Takeda H., Koga Y. (2009). Clinical effects of kestose, a prebiotic oligosaccharide, on the treatment of atopic dermatitis in infants. Clin. Exp. Allergy.

[64] Siziba L.P., Mank M., Stahl B., Kurz D., Gonsalves J., Blijenberg B., Rothenbacher D., Genuneit J. (2022). Human milk oligosaccharide profiles and child atopic dermatitis up to 2 years of age: The Ulm SPATZ Health Study. Pediatr. Allergy Immunol..

[65] Zou X., Zou X., Gao L., Zhao H. (2024). Gut microbiota and psoriasis: Pathogenesis, targeted therapy, and future directions. Front. Cell. Infect. Microbiol..

[66] Buhaș M.C., Gavrilaș L.I., Candrea R., Cătinean A., Mocan A., Miere D., Tătaru A. (2022). Gut microbiota in psoriasis. Nutrients.

[67] Wang Q., Wang J., Sun X., Liu L., Zhang M., Yu Y., Gao P., Hong S., Li X. (2025). Evidence-based dietary recommendations for patients with psoriasis: A systematic review. Clin. Nutr..

[68] Xue M., Deng Q., Deng L., Xun T., Huang T., Zhao J., Wei S., Zhao C., Chen X., Zhou Y. (2025). Alterations of gut microbiota for the onset and treatment of psoriasis: A systematic review. Eur. J. Pharmacol..

[69] Kapoor B., Gulati M., Rani P., Gupta R. (2022). Psoriasis: Interplay between dysbiosis and host immune system. Autoimmun. Rev..

[70] Zhao Y., Yu C., Zhang J., Yao Q., Zhu X., Zhou X. (2025). The gut-skin axis: Emerging insights in understanding and treating skin diseases through gut microbiome modulation. Int. J. Mol. Med..

[71] Pachauri A., Sharma S. (2025). Unravelling the gut-skin axis: The role of gut microbiota in pathogenesis and management of psoriasis. Inflammopharmacology.

[72] Gong X., Zhang Z., Shi X., Zhu Y., Ali F., Dong Y., Zhang F., Zhang B. (2024). Structural elucidation and anti-psoriasis activity of a novel polysaccharide from *Saussurea costus*. Carbohydr. Polym..

[73] Zeng B., Yan Y., Zhang Y., Wang C., Huang W., Zhong X., Chen Z., Xie M., Yang Z. (2024). *Dendrobium officinale* polysaccharide (DOP) inhibits cell hyperproliferation, inflammation and oxidative stress to improve keratinocyte psoriasis-like state. Adv. Med. Sci..

[74] Chen M., Peng Y., Zhu R., Luo X., Yang X., Chen J., Chen H., Zhou W., Du Z. (2025). Therapeutic potential of *Rosa rugosa* polysaccharide and its nanofiber membrane in psoriasis via PI3K-AKT/mTOR pathway inhibition. Int. J. Biol. Macromol..

[75] Leng H., Pu L., Xu L., Shi X., Ji J., Chen K. (2018). Effects of aloe polysaccharide, a polysaccharide extracted from *Aloe vera*, on TNF-α-induced HaCaT cell proliferation and the underlying mechanism in psoriasis. Mol. Med. Rep..

[76] Liu S., Song S., Zhang Y., Yan L., Chen Y., Li W., Jalil B., Wu C., Fu Y., Chen X. (2025). Delivery of penetration-enhancing antioxidant polyphenol nanoparticles with *Codonopsis pilosula* polysaccharide microneedles for synergistic treatment of psoriasis. Carbohydr. Polym..

[77] Li Y., Lou Y., Chen Y., Yang J., Li D., Jiang B., Lan J., Wen J., Fu Y., Zhang Y. (2021). Polysaccharide mycophenolate-based nanoparticles for enhanced immunosuppression and treatment of immune-mediated inflammatory diseases. Theranostics.

[78] Li X.-L., Wang Z.-H., Zhao Y.-X., Luo S.-J., Zhang D.-W., Xiao S.-X., Peng Z.-H. (2012). Purification of a polysaccharide from *Gynostemma pentaphyllum* Makino and its therapeutic advantages for psoriasis. Carbohydr. Polym..

[79] Fahlquist-Hagert C., Sareila O., Rosendahl S., Holmdahl R. (2022). Variants of beta-glucan polysaccharides downregulate autoimmune inflammation. Commun. Biol..

[80] Chen R.X., Zheng S., Guo C.Y., Zhang Q. (2022). Effects of Astragalus polysaccharide on imiquimod-induced psoriasiform dermatitis in mice and its mechanisms. Zhongguo Ying Yong Sheng Li Xue Za Zhi.

[81] Buhaș M.C., Candrea R., Gavrilaș L.I., Miere D., Tătaru A., Boca A., Cătinean A. (2023). Transforming psoriasis care: Probiotics and prebiotics as novel therapeutic approaches. Int. J. Mol. Sci..

[82] Moludi J., Fathollahi P., Khedmatgozar H., Tabrizi F.P.F., Zare A.G., Razmi H., Amirpour M. (2022). Probiotics supplementation improves quality of life, clinical symptoms, and inflammatory status in patients with psoriasis. J. Drugs Dermatol..

[83] Abdi A., Oroojzadeh P., Valivand N., Sambrani R., Lotfi H. (2024). Immunological aspects of probiotics for improving skin diseases: Influence on the gut-brain-skin axis. Biochem. Biophys. Res. Commun..

[84] Hu J., Yao W., Chang S., You L., Zhao M., Cheung P.C.-K., Hileuskaya K. (2022). Structural characterization and anti-photoaging activity of a polysaccharide from *Sargassum fusiforme*. Food Res. Int..

[85] Tao X., Hu X., Wu T., Zhou D., Yang D., Li X., Fu Y., Zheng F., Yue H., Dai Y. (2023). Characterization and screening of anti-melanogenesis and anti-photoaging activity of different enzyme-assisted polysaccharide extracts from *Portulaca oleracea* L.. Phytomedicine.

[86] Yang M., Tao L., Wang Z., Li L., Luo J., Pai K., Li W., Zhao C., Sheng J., Tian Y. (2023). The mechanism of peach gum polysaccharide preventing UVB-induced skin photoaging by regulating matrix metalloproteinases and oxidative factors. Molecules.

[87] Li Y., Ma H., Shi L., Zhang Z., Wu Y., Yan C., Chen Y., Lu Y. (2025). Purification, characterization and protective effects on UVB-induced photoaging in zebrafish of *Pleurotus citrinopileatus* polysaccharide PCP-2. J. Sci. Food Agric..

[88] Li Y., Mei M., Wang Q., Gen L., Hao K., Zhong R., Mo T., Jiang J., Zhu W. (2024). Structural characteristics and anti-photoaging effect of *Pyracantha fortuneana* fruit polysaccharides in vitro and in vivo. Int. J. Biol. Macromol..

[89] Zhang Y., Zheng S., Si H., Liu Y., Xie F., Wang X., Wu S., Chen B., Zhai C., Qiao Y. (2025). Structure characterization and protective effect against UVB irradiation of polysaccharides isolated from peach gums. Int. J. Biol. Macromol..

[90] Fan L., Luan X., Jia Y., Ma L., Wang Z., Yang Y., Chen Q., Cui X., Luo D. (2024). Protective effect and mechanism of *Lycium barbarum* polysaccharide against UVB-induced skin photoaging. Photochem. Photobiol. Sci..

[91] Guo L., Yang Y., Pu Y., Mao S., Nie Y., Liu Y., Jiang X. (2024). *Dendrobium officinale* Kimura & Migo polysaccharide and its multilayer emulsion protect skin photoaging. J. Ethnopharmacol..

[92] Wei K., He L., Li X., Wu M., Wang H., Hu L., Xu H., Zhang Y., Zhou L., Xu X. (2025). The β-glucan from *Lentinus* alleviates UVB-induced dermal fibroblast senescence and skin photoaging. Int. J. Biol. Macromol..

[93] Chen Q., Li M., Li Z., Mei M., Lin Y., Shu P., Zhu W. (2025). *Alhagi camelorum* seed polysaccharide alleviates methylglyoxal-induced skin damage via antioxidant and anti-inflammatory actions. Int. J. Biol. Macromol..

[94] Lin H., Cheng K.-C., Lin J.-A., Hsieh L.-P., Chou C.-H., Wang Y.-Y., Lai P.-S., Chu P.-C., Hsieh C.-W. (2022). *Pholiota nameko* polysaccharides protect against ultraviolet A-induced photoaging by regulating matrix metalloproteinases in human dermal fibroblasts. Antioxidants.

[95] Lai Y., Wang Y., Mueed A., Shu P., You L., Zhong J. (2026). Anti-photoaging effects of a polysaccharide from *Kappaphycus alvarezii* in vitro and in vivo. Mar. Drugs.

[96] Kim Y.-I., Oh W.-S., Song P.H., Yun S., Kwon Y.-S., Lee Y.J., Ku S.-K., Song C.-H., Oh T.-H. (2018). Anti-photoaging effects of low-molecular-weight fucoidan on ultraviolet B-irradiated mice. Mar. Drugs.

[97] Cheong K.L., Chen Q., Aweya J.J., Ji X.L., Zhong S., Tan K. (2025). Trends in polysaccharide-based hydrogels for skin anti-aging and skin antioxidant. Int. J. Biol. Macromol..

[98] Wu S., Liu G., Shao P., Lin X., Yu J., Chen H., Li H., Feng S. (2024). Transdermal sustained release properties and anti-photoaging efficacy of liposome-thermosensitive hydrogel system. Adv. Healthc. Mater..

[99] Akhter K.F., Mumin M.A., Lui E.M.K., Charpentier P.A. (2021). Transdermal nanotherapeutics: *Panax quinquefolium* polysaccharide nanoparticles attenuate UVB-induced skin cancer. Int. J. Biol. Macromol..

[100] Yue Y., Fang Y., Jia R., Cao K., Chen X., Xia H., Cheng Z. (2023). Study on the antioxidant effect of shikonin-loaded β-cyclodextrin forming host–guest complexes that prevent skin from photoaging. Int. J. Mol. Sci..

[101] Rudtanatip T., Somintara S., Sakaew W., El-Abid J., Cano M.E., Jongsomchai K., Wongprasert K., Kovensky J. (2022). Sulfated galactans from *Gracilaria fisheri* with supplementation of octanoyl promote wound healing activity in vitro and in vivo. Macromol. Biosci..

[102] Tan G., Wang L., Pan W., Chen K. (2022). Polysaccharide electrospun nanofibers for wound healing applications. Int. J. Nanomed..

[103] Abazari M., Akbari T., Hasani M., Sharifikolouei E., Raoufi M., Foroumadi A., Sharifzadeh M., Firoozpour L., Khoobi M. (2022). Polysaccharide-based hydrogels containing herbal extracts for wound healing applications. Carbohydr. Polym..

[104] Zheng B.D., Xiao M.T. (2023). Polysaccharide-based hydrogel with photothermal effect for accelerating wound healing. Carbohydr. Polym..

[105] Chinta M.L., Gandam P.K., Sivasankar M.V., Parcha S.R. (2025). Tamarind (*Tamarindus indica* L.) seed polysaccharide: A promising biopolymer for drug delivery, wound healing, tissue engineering and beyond. Carbohydr. Res..

[106] Zhao B., Zhang X., Han W., Cheng J., Qin Y. (2017). Wound healing effect of an *Astragalus membranaceus* polysaccharide and its mechanism. Mol. Med. Rep..

[107] Wen W., Yang L., Wang X., Zhang H., Wu F., Xu K., Chen S., Liao Z. (2023). Fucoidan promotes angiogenesis and accelerates wound healing through AKT/Nrf2/HIF-1α signalling pathway. Int. Wound J..

[108] Ahmadian Z., Jelodar M.Z., Rashidipour M., Dadkhah M., Adhami V., Sefareshi S., Ebrahimi H.A., Ghasemian M., Adeli M. (2023). A self-healable and bioadhesive acacia gum polysaccharide-based injectable hydrogel for wound healing acceleration. DARU J. Pharm. Sci..

[109] Tang L., Xie S., Wang D., Wei Y., Ji X., Wang Y., Zhao N., Mou Z., Li B., Sun W.R. (2025). Astragalus polysaccharide/carboxymethyl chitosan/sodium alginate based electroconductive hydrogels for diabetic wound healing and muscle function assessment. Carbohydr. Polym..

[110] Lin J., Wang L., Li W., Li Y., Tang F., Xu J., Li W., Gong H., Jiang X., Feng Y. (2024). Dried tangerine peel polysaccharide accelerates wound healing by recruiting anti-inflammatory macrophages. Int. Immunopharmacol..

[111] Zhang L., Yang J., Liu W., Ding Q., Sun S., Zhang S., Wang N., Wang Y., Xi S., Liu C. (2023). A *Phellinus igniarius* polysaccharide/chitosan-arginine hydrogel for promoting diabetic wound healing. Int. J. Biol. Macromol..

[112] Jiang F., Ding Y., Tian Y., Yang R., Quan M., Tong Z., Zhang X., Luo D., Chi Z., Liu C. (2022). Hydrolyzed low-molecular-weight polysaccharide from *Enteromorpha prolifera* exhibits high anti-inflammatory activity and promotes wound healing. Biomater. Adv..

[113] Hao Y., Wang J., Zhang H., Liu Q., Wang X., Wei Y., Liang Z., Hu Y., Huang D. (2025). Konjac glucomannan/*Bletilla striata* polysaccharide composite hydrogel: A promising anti-inflammatory dressing for accelerated wound healing. Carbohydr. Polym..

[114] Li F., Liu T., Liu X., Han C., Li L., Zhang Q., Sui X. (2024). *Ganoderma lucidum* polysaccharide hydrogel accelerates diabetic wound healing by regulating macrophage polarization. Int. J. Biol. Macromol..

[115] Li W., Yang J., Kong W., Fan P., Guan D., Bao Y., Wu G., Wang S., Sun Y. (2025). A polysaccharide-based self-gelling powder with antibacterial and antioxidant capacities for acute hemostasis and efficient infected wound healing. Adv. Healthc. Mater..

[116] Zou Y., Yang Y., Pei J., Sun P., Wang Y. (2025). *Ganoderma lucidum* polysaccharide/carboxymethyl chitosan hydrogels modulate macrophage polarization for wound healing. Biomacromolecules.

